# Predicting Survival in Veterans with Follicular Lymphoma Using Structured Electronic Health Record Information and Machine Learning

**DOI:** 10.3390/ijerph18052679

**Published:** 2021-03-07

**Authors:** Chunyang Li, Vikas Patil, Kelli M. Rasmussen, Christina Yong, Hsu-Chih Chien, Debbie Morreall, Jeffrey Humpherys, Brian C. Sauer, Zachary Burningham, Ahmad S. Halwani

**Affiliations:** 1 Veritas, Division of Epidemiology, School of Medicine, University of Utah, Salt Lake City, UT 84112, USA; vikas.patil@hsc.utah.edu (V.P.); kelli.rasmussen@hsc.utah.edu (K.M.R.); c.m.yong@utah.edu (C.Y.); sophia.hsuchih.chien@utah.edu (H.-C.C.); u0058546@gcloud.utah.edu (D.M.); jeff.humpherys@hsc.utah.edu (J.H.); brian.sauer@utah.edu (B.C.S.); Zach.Burningham@hsc.utah.edu (Z.B.); ahmad.halwani@hsc.utah.edu (A.S.H.); 2 George E. Wahlen Veterans Health Administration, Salt Lake City, UT 84148, USA; 3 Hematology & Hematologic Malignancies, Huntsman Cancer Institute, Salt Lake City, UT 84112, USA

**Keywords:** machine learning, prognosis, follicular lymphoma, survival analysis, random survival forest, predictive analytics, veterans health administration, electronic health records, healthcare, medical and health data

## Abstract

The most accurate prognostic approach for follicular lymphoma (FL), progression of disease at 24 months (POD24), requires two years’ observation after initiating first-line therapy (L1) to predict outcomes. We applied machine learning to structured electronic health record (EHR) data to predict individual survival at L1 initiation. We grouped 523 observations and 1933 variables from a nationwide cohort of FL patients diagnosed 2006–2014 in the Veterans Health Administration into traditionally used prognostic variables (“curated”), commonly measured labs (“labs”), and International Classification of Diseases diagnostic codes (“ICD”) sets. We compared performance of random survival forests (RSF) vs. traditional Cox model using four datasets: curated, curated + labs, curated + ICD, and curated + ICD + labs, also using Cox on curated + POD24. We evaluated variable importance and partial dependence plots with area under the receiver operating characteristic curve (AUC). RSF with curated + labs performed best, with mean AUC 0.73 (95% CI: 0.71–0.75). It approximated, but did not surpass, Cox with POD24 (mean AUC 0.74 [95% CI: 0.71–0.77]). RSF using EHR data achieved better performance than traditional prognostic variables, setting the foundation for the incorporation of our algorithm into the EHR. It also provides for possible future scenarios in which clinicians could be provided an EHR-based tool which approximates the predictive ability of the most accurate known indicator, using information available 24 months earlier.

## 1. Introduction

Follicular lymphoma (FL), the most common indolent non-Hodgkin lymphoma [1], accounts for about 20% of non-Hodgkin lymphoma [2,3]. Patients with FL have highly heterogeneous prognoses; some patients experience an indolent course of disease, while others endure a more aggressive disease with a trajectory that can include frequent progression, relapse, and early demise [4,5,6]. FL treatments can be associated with morbidity and, rarely, mortality [7,8]. Thus, patients and clinicians must calibrate therapy choice to the risk posed by FL. Otherwise, treatment risks could outweigh the benefits [4,9]. In order to apply risk-adapted treatment strategies effectively, clinicians must be able to accurately identify high-risk patients early in the course of disease, but the methods currently available for this task have significant drawbacks.

Specific patient-, disease-, and environment-related variables that can serve as prognostic factors in oncology play a pivotal role in understanding disease trajectory, designing clinical trials, making treatment decisions, and providing individual patients with accurate information about their health risks [10]. The most commonly used FL prognostic index is the Follicular Lymphoma International Prognostic Index (FLIPI) [11], which resulted from an international collaboration that collected clinical characteristics and associated outcomes from a large number of lymphoma patients treated with chemotherapy on various clinical trial protocols. The variables included in the final model were age (>60 years), disease stage (III–IV), hemoglobin (<12 g/dL), number of nodal areas (>4), and lactate dehydrogenase (LDH) level (>upper limit of normal). A study validating FLIPI [12] obtained a Harrell’s concordance index (C-index) of 0.66. As with FLIPI, most traditional prognostic indices in oncology have typically been based on human-abstracted variables collected during clinical trials [13,14]. These indices were optimized not only for predictive performance, but also for ease of use by clinicians at the point of care, constraining the number of variables that could be included and the complexity of score calculations. FLIPI’s optimization for ease of recollection and calculation in a pre-electronic health record (EHR) era makes it poorly suited for current clinical practice in the era of near-universal EHR use.

In recent years, disease progression within two years following initiation of first-line (L1) treatment with chemoimmunotherapy (aka progression of disease within 24 months [POD24]) [6,15] has risen to prominence as a simple and relatively accurate way of defining a high-risk group of patients with decreased overall survival. Patients whose response to L1 treatment lasts beyond two years demonstrate survival rates similar to those of the age-matched general population [15]. However, using POD24 to assess risk requires waiting two years after the start of L1 treatment in order to identify these patients; during that time, neither patient nor physician can be sure about the disease prognosis. Furthermore, using POD24 does not support a risk-adapted strategy in L1 treatment, whereas, ideally, patients and clinicians could take into account a patient’s risk status while choosing an L1 therapy.

EHR systems, now widespread, could theoretically automatically extract and calculate prognostic indices and present the resulting risk prediction to practicing physicians. Absent the need to provide for easy recollection and calculation by clinicians, EHR-based indices could instead be optimized for predictive performance and ease of extraction. Such an optimization would favor the use of structured or semi-structured information over information presented in unstructured narratives.

For survival analysis in oncology, the traditional Cox proportional hazard model investigates the relationship between survival time and one or more variables. Although well established and easily interpretable, the Cox model carries key limitations. It must satisfy linearity and proportional hazards assumptions and can be applied only to a restricted number of predictors, thus limiting its ability to account for interactions between variables. Compared with the Cox model, machine learning can better manage large numbers of predictors—even handling cases with more predictors than observations—and can account for both non-linearity and variable interactions [16]. The random survival forest (RSF) method, a flexible, non-parametric ensemble tree machine learning approach extended from Breiman’s [17] random forest method in order to analyze right-censored survival data [18], shows promise as a way to use high-dimensional, structured EHR data to identify factors for predicting patient risk [19]. RSF’s reliance on only the data in seeking a predictive model offers real advantages: it does not require model assumptions, and it can be applied in exploratory investigations, allowing the discovery of unexpected risk factors even with a paucity of prior survival data [18,20]. RSF avoids overfitting [21], while effectively handling outliers [22]. Its out-of-bag prediction also provides reliable inferences for training data, and it can measure relative contributions of different variables (i.e., variable importance) to the survival prediction [20].

Machine learning techniques have been widely used in cancer research [23,24,25]. However, to our knowledge, no prior study has used RSF to predict patients’ prognoses in FL and investigated how structured and semi-structured EHR data could benefit prediction.

In this study, we applied RSF to predict FL patients’ survival and risks using EHR data from a cohort of patients treated within the Veterans Health Administration (VHA). Our findings supported our hypothesis that this method would outperform traditional approaches and achieve predictive performance similar to that of POD24. Our results provide clinicians with insights into the relative importance of traditional and new variables in predicting FL patients’ risk.

## 2. Materials and Methods

This research was approved by the University of Utah Institutional Review Board (#00083982) and the VA Salt Lake City Human Research Protection Program.

### 2.1. Cohort Selection

**Figure 1:**
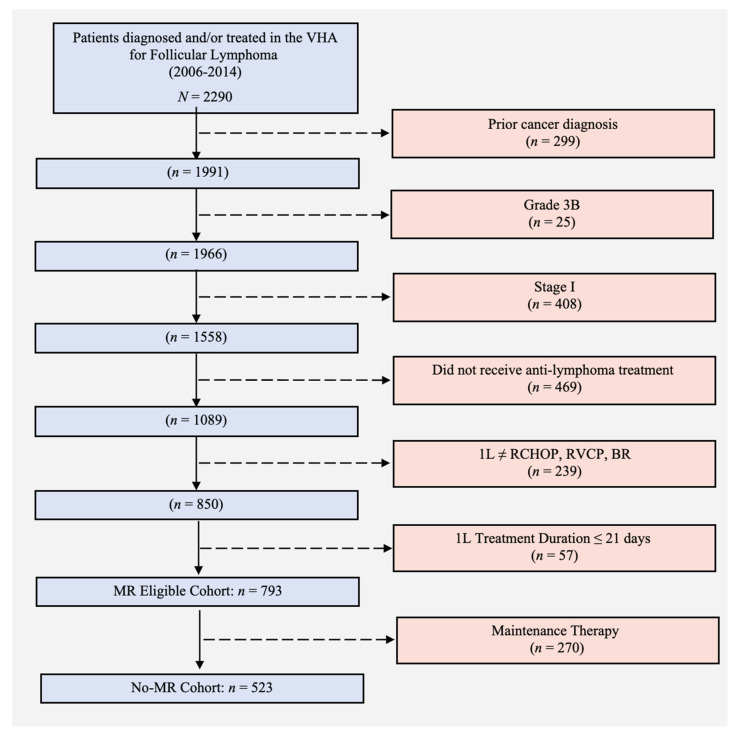
Study cohort attrition. 1L: first-line treatment; BR: bendamustine and rituximab; MR: maintenance rituximab; RCHOP: rituximab, cyclophosphamide, doxorubicin, vincristine, and prednisone; RCVP: rituximab, cyclophosphamide, vincristine, and prednisone; VHA: Veterans Health Administration.

Using data from the Veterans Affairs (VA) Cancer Registry System and pharmacy dispensation records from the VA Corporate Data Warehouse, we identified a nationwide cohort of patients with grade 1–3a, stage II–IV FL diagnosed from 1 January 2006 to 31 December 2014 in the VHA, who received any of three widely used L1 therapies—rituximab combined with cyclophosphamide, doxorubicin, vincristine, and prednisone; rituximab combined with cyclophosphamide, vincristine, and prednisone; or bendamustine combined with rituximab [26]. Table 1 shows patients’ characteristics by treatment. We excluded patients who received maintenance therapy (see Appendix A for rationale), as well as patients without a hematology/oncology visit within 6 months of diagnosis, as these latter patients likely received treatment outside the VHA which we would have limited ability to identify. Patients with a VA Cancer Registry System record of another malignancy prior to FL diagnosis were also excluded. The final cohort included 523 patients. Figure 1 shows the study flow diagram.

**Figure 2:**
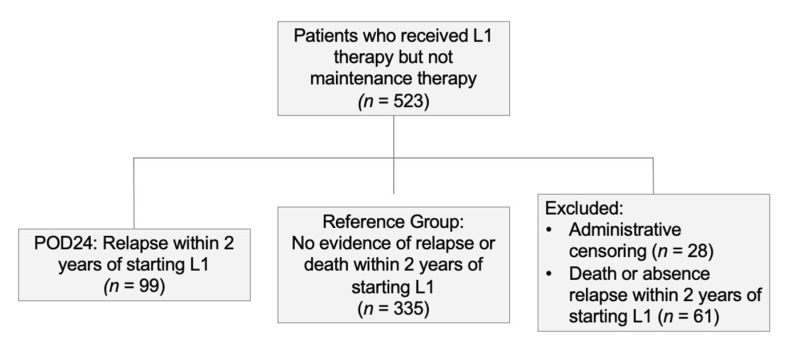
Participant selection diagram for Cox model with curated + POD24 dataset. L1: first-line treatment; POD24: progression of disease within 24 months of initiation of first-line treatment.

The dataset included 523 patients, with 1933 variables. Median follow-up time was 5.03 years, and median survival time 9.32 years. At five years, 150 (28.68%) patients had died, 111 (21.22%) patients had been administratively censored at the end of the study observation period, and 262 (50.10%) patients were alive. Within two years, 99 patients (22.81%) experienced progression of disease (POD24). As in Casulo et al. [15], we excluded from our POD24 analysis 89 patients who were administratively censored (*n* = 28) or died (*n* = 61) from causes unrelated to their disease relapse, to the best of our knowledge. Figure 2 illustrates cohort distribution for POD24 and reference groups.

### 2.2. Feature Extractions

We grouped covariates into three groups:

- A curated clinical set (“curated”) comprised patient demographics and disease-specific characteristics commonly recognized to be associated with survival, which were available in structured form in the VA Cancer Registry System or Corporate Data Warehouse (Table A1). Patient characteristics included age, sex, and modified Charlson comorbidity index; disease-specific characteristics included stage, grade, and lactate dehydrogenase at L1 initiation. We also included treatment used for L1.
- The second group (“labs”) included results of 33 lab values typically obtained prior to initiation of L1, extracted from the EHR lab domain. These data included most of the labs (available in 70% or more of patients) in the complete blood count and comprehensive metabolic panels (Table A1). We included medians and ranges of lab results for the period starting three months prior to start of L1 and ending just prior to start of L1. RSF handles missing data itself; for the Cox model, missing data were imputed by random forest imputation algorithm [27] using randomForestSRC R package [28].
- Finally, a larger group of variables (“ICD”) included any International Classification of Diseases (ICD) diagnostic codes present from one year prior to L1 initiation to three months prior to L1, with information indicating presence or absence of ICD codes as well as how many times each individual code was present during this nine-month period. There were 1841 ICD codes overall.

### 2.3. Outcomes

Vital status and date of death for patients who died before the end of observation period on 31 December 2018 were obtained from the Corporate Data Warehouse, which aggregates vital status from multiple sources. Survival was measured from first dispensation of L1 to date of death as recorded in Corporate Data Warehouse records. Patients were censored if they were recorded to have another cancer in the VA Cancer Registry System, or at the end of observation period. Five-year overall survival is an established clinical milestone in FL studies [29,30,31] and POD24 predicts a high-risk group with a median survival of five years; we compared Cox and RSF methods’ relative ability to predict five-year survival. We defined as belonging to the high-risk group any patient who died within five years after L1 initiation, with the low-risk group defined as patients who lived beyond the five-year time point.

### 2.4. Models

The Cox model estimates the hazard function *h(t)*, which gives instantaneous risk of event, by a linear function (i.e., the log risk of failure is a linear combination of covariates [see (2) below]). Given the hazard function, survival function in terms of time, the probability that an observation survives beyond a specific time, can be obtained. The model parameters are estimated through optimization of the cox partial likelihood.

The hazard at time *t* given covariates $x=\;(x_{0},\;x_{1},\;\ldots ,\;x_{p})$ is defined as

(1) $$h\left(t|x\right)=\;h_{0}\left(t\right)\exp\left(\sum_{j}^{p}x_{j}\beta_{j}\right)$$

From (1), we can also obtain

(2) $$\ln\left(h\left(t|x\right)\right)=\;\ln(h_{0}\left(t\right))+\;\left(\sum_{j}^{p}x_{j}\beta_{j}\right).$$

where $h_{0}\left(t\right)$ is the baseline hazard function and does not need to be specified while estimating the parameters $\beta \;=\;{\left(\beta_{0},\beta_{1},\;\ldots ,\;\beta_{2}\right)}^{T}$ through maximizing the partial likelihood defined as the following formula:

(3) $$L\left(\beta \right)=\;\underset{f\in F}{\prod }\frac{\exp\left(\beta^{T}x^{j_{f}}\right)}{\sum_{j\in R}\exp\left(\beta^{T}x^{j}\right)\;}$$

where *F* is the set of indices of failures, *R* is the set of indices of individuals at risk at time *t*, and $j_{f}$ is the index of failure at time *t*.

For individual risk prediction in Cox model, the estimated hazard for individual *i*, with covariate vector $x_{i}$ follows:

(4) $$\hat{h_{i}}\left(t\right)=\;\hat{h_{0}}\left(t\right)\exp\left(x_{i}^{\prime }\hat{\beta }\;\right),$$

where $\hat{\beta }$ is the estimated coefficients by maximizing partial likelihood, $\hat{h_{i}}\left(t\right)$ is the estimated hazard for individual i and $\hat{h_{0}}\left(t\right)$ is the estimated baseline hazard.

Similarly, the survival function of individual *i* at time t is then is calculated with the following formula:

(5) $$\hat{S_{i}}\left(t\right)=\;\hat{S_{0}}\left(t\right)\exp\left(x_{i}^{\prime }\hat{\beta }\right),$$

with $\hat{S_{0}}\left(t\right)=\exp\left(-\hat{\Lambda_{0}}\left(t\right)\right)$ and $\hat{\Lambda_{0}}\left(t\right)$ is the cumulated hazard until time *t* [32].

However, the Cox model assumes that the data have linear proportional hazard, which assumption, in many cases—especially for high dimensional data with non-linear interactions—is not satisfied. Therefore, a more complicated non-linear model, such as a regularized Cox model or a machine learning model, is needed.

We used a regularized Cox model with lasso approach when the dimensionality of the data increases. The lasso regularization method is a variable selection and shrinkage method in Cox model, where the log partial likelihood is minimized subject to the sum of the absolute values of the parameter bounded by a given constant [33]. The constraint shrinks the coefficients; some are shrunk to zero, thus reducing the estimation variance [33]. We used a five-fold cross-validation approach to obtain the optimal penalty parameter [34].

Among machine learning approaches, RSF is a flexible non-parametric ensemble tree method, extended from Breiman’s [17] random forest method for analyzing right-censored survival data [18]. Random forest is a popular tree-based ensemble method and each tree is built on randomly selected variables [35]. Bootstrap samples of the data are selected to build the trees. Each node of the tree is split using a randomly selected set of variables. The randomization decorrelates the trees and keeps the variance relatively small, since combining the predictions of all these uncorrelated trees built with different samples and subsets of features leads to lower variance [17]. The trees are grown to the full extent, which reduces bias. The forest prediction is based on the majority of votes, with each tree receiving one vote for the observation that is not in the bootstrap sample (out-of-bag observation).

RSF is extended from the random forest method. For the RSF approach,

1. Bootstrap samples of the training data are selected to build the trees. For each bootstrap sample, about 2/3 of the observations are selected and 1/3 are left out.
2. In each bootstrap sample, a survival tree is constructed. Each node, *p* candidate variables are randomly selected to build the tree. The split of the nodes maximizes the survival difference between daughter nodes; in this study, a log-rank splitting rule is used to determine the split of the nodes [18].
3. The tree is grown to full size under the constraint that there should be at least one event with unique survival times at each terminal node.
4. Survival curves are estimated for the out-of-bag observations and the average survival curves are calculated as the survival curve for each subject. The cumulative hazard functions in terminal nodes are time-dependent. Performance is assessed based on the testing set using the RSF model obtained from the training and parameter tuning process.

The nodes of the trees are split using a survival criterion considering survival time and censoring status information [18]. The cumulative hazard function is an ensemble estimate of all the trees.

To train each model, patients were randomly split into a training set (80%) for model training with five-fold cross-validation approach and a testing set (20%) for performance evaluation. Optimal parameters for RSF were selected using grid search [36] with the cross-validation process. The details of parameter tuning process are provided in Appendix B. To calculate average performance and 95% confidence intervals (CI), we repeated this process 30 times. The mean AUC is the mean of the 30 C-indices and AUCs. The baseline characteristic summarization, as well as variable importance and partial dependence plots, are shown based on the split with performance closest to the average of 30 splits [37].

For each approach, we fit models with four different sets of input variables with increasing granularity: curated set, curated+ labs set, curated + ICD set, and curated + labs + ICD set. Only the curated set satisfied proportional hazard and linearity assumptions, i.e., had survival curves for two strata with hazard functions are roughly proportional over time for each categorical covariate, and the relationship between the ln(hazard of death) and each of the continuous variables in the model were close to linear after adjusting other covariates. No interactions were fit or tested. A traditional Cox model is unable to model the curated + ICD and curated + labs + ICD sets due to the high dimensionality, so a “regularized” Cox model with lasso approach [34] was used for these two sets. We also used the regularized Cox model for the curated + labs set where the Cox model assumptions were violated.

### 2.5. Use of RSF to Predict High or Low Risk

We set the survival function S(t) as the probability that a patient would survive beyond five years after the start of L1. We used predictSurvProb function in the pec R package [38] for the survival probability calculation [39]. When S(t) < an optimal cut-off point, where the optimal cut-off point was identified using Youden’s index (J), defined as J = max{sensitivity + specificity − 1} [40], we classified a patient as belonging to the high-risk group; otherwise, we categorized the patient as being at low risk.

We used training data to “grow” the RSF, in which each patient in the test set was passed down the trees. Individual survival functions for each patient were extracted by evaluating the obtained cumulative hazard functions over a five-year time interval. High- and low-risk groups were then determined by the individual survival functions with the optimal cut-off point.

### 2.6. Model Performance Measures

We evaluated the predictive performance of the models using area under the receiver operating characteristic curve (AUC), which is also referred to as a ‘C-statistic’ or C-index [41], a rank-order statistic for assessing predictions against true outcomes (defined as the ratio of the concordant pairs to the total comparable pairs [42]), served as a standard performance measure for model assessment in survival analysis. AUC provides information about how well a model can distinguish between classifications, where a higher AUC reflects a better model.

### 2.7. Model Interpretation

We used variable importance and partial dependence plots to explain the best-performing models, measuring a given variable’s importance by increase or decrease in prediction error after dropping the predictor variable at RSF nodes. Friedman 2001 [43] proposed the partial dependence plot to interpret machine learning algorithms; the plot illustrates the average change of the predicted value when the variable changes over its marginal distribution. The partial dependence plot serves as a useful tool in understanding the relationship between predictors and predictions in various predictive settings [44].

## 3. Results

We used R [45] to perform the analyses. Model performances on four datasets are shown in Table 2 and Table 3. Using the curated clinical variable set, the baseline Cox model yielded a mean AUC of 0.64 (95% CI: 0.61–0.67), while the RSF model achieved a mean AUC of 0.67 (95% CI: 0.65–0.70). The addition of lab results had little effect on the performance of the Cox model, with a mean AUC of 0.62 (95% CI: 0.60–0.64). Using a regularized Cox model for curated + labs set boosted AUC to 0.71 (95% CI: 0.69–0.73). With more variables available (ICD codes), a regularized Cox model achieved comparable performance to RSF. The variables retained in the regularized Cox model are reported in Appendix C. The RSF model showed marked improvement in prediction performance on the curated + labs set, with a mean AUC of 0.73 (95% CI: 0.71–0.75). The curated + labs set with RSF model turned out to be the best-performing combination of dataset and approach. We found no significant difference between RSF performance on the curated + labs set (95% CI for AUC: 0.71–0.75) and curated + ICD + labs set (95% CI for AUC: 0.65–0.73). RSF performance on curated + ICD + labs showed much higher variance than performance on the curated + labs dataset. The best-performing combination of approach and dataset—the RSF model with curated + labs set—achieved a performance that was close to but unable to beat that of a Cox model based on POD24 (mean AUC 0.74 [95% CI: 0.71–0.77]).

**Figure 3:**
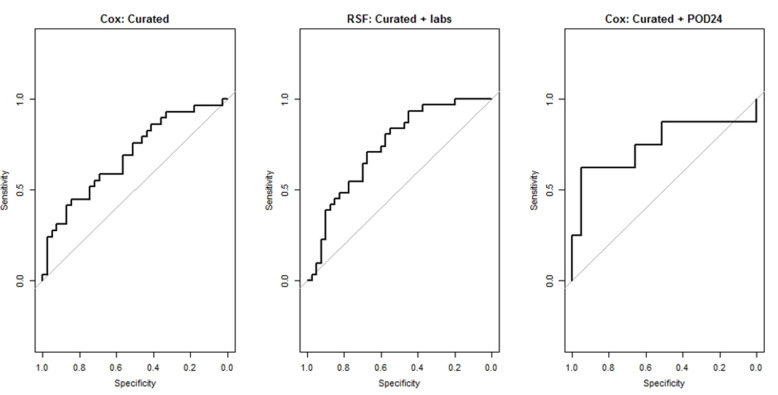
ROC plots for Cox applied to curated dataset, RSF applied to curated + labs set, and Cox applied to curated + POD24 set. ROC: receiver operating characteristic; RSF: random survival forest; POD24: progression of disease within 24 months of initiation of first-line treatment.

Receiver operating characteristic (ROC) curves are presented in Figure 3 for the Cox model applied to curated set, RSF model applied to the curated + labs set, and Cox model applied to the curated + POD24 dataset. The ROC curves show the tradeoff between sensitivities and specificities for each model.

**Figure 4:**
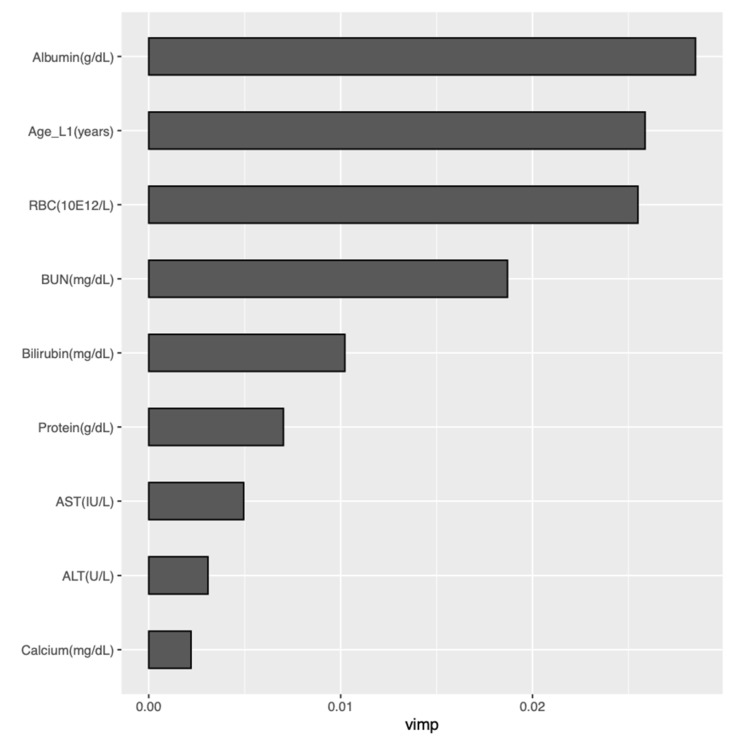
Variable importance plot for RSF analysis of the curated + labs dataset. RSF: random survival forest; L1: time of initiation of first-line therapy; RBC: erythrocytes: BUN: urea nitrogen; AST: aspartate aminotransferase; ALT: alanine aminotransferase; vimp: variable importance.

**Figure 5:**
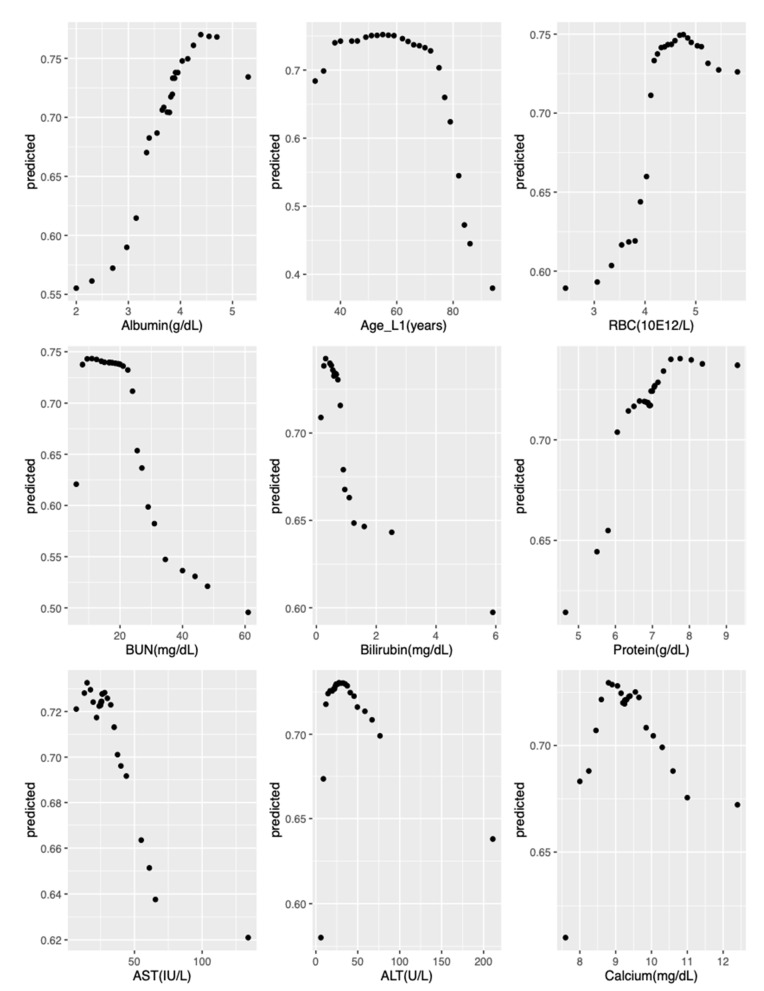
Partial-dependence plot for the top nine variables in the RSF model applied to curated + labs dataset. Vertical axis shows predicted five-year survival probability. RSF: random survival forest; L1: time of initiation of first-line therapy; RBC: erythrocytes: BUN: urea nitrogen; AST: aspartate aminotransferase; ALT: alanine aminotransferase.

Figure 4 provides variable importance plots for the RSF model applied to the curated + labs set. The nine most influential predictors were examined with partial dependence plots to explore their relationship with predicted survival probabilities. These nine most important variables, in order of decreasing influence, were albumin, age at L1 initiation, erythrocytes, urea nitrogen, bilirubin, protein, aspartate aminotransferase, alanine aminotransferase, and calcium. Figure 5 provides partial dependence plots for these nine variables. The vertical axis shows the predicted survival probability, and the horizontal axis indicates change in predictor values. The partial dependence plots illustrate that the predicted survival probabilities decrease with age above 60 years, dropping faster with age over 70 years. Similarly, survival decreased as albumin and erythrocytes decreased and urea nitrogen, bilirubin, protein, aspartate aminotransferase, alanine aminotransferase, and calcium increased beyond their respective normal ranges, indicating worsening renal (urea nitrogen), liver (albumin, bilirubin, alanine aminotransferase), and marrow (erythrocyte, hemoglobin) functions.

Our best-performing model and data set, RSF with curated + labs set, identified patients at elevated risk of death within five years (hazard ratio: 4.39 [95% CI: 2.11–9.14]) (Table 4) and with greater likelihood to show factors traditionally considered indicative of high risk (hemoglobin, lactate dehydrogenase), even though these variables were not among the top variables used by the RSF algorithm to predict risk. Lactate dehydrogenase and hemoglobin were the only traditional indicators found to be statistically significant risk factors (*p*-value < 0.05). Table 5 provides baseline characteristics of predicted high- and low-risk groups. Table 4 shows how the high-/low-risk classifications for RSF at the beginning of L1 compared to the classifications for POD24 two years later. The estimated hazard ratio for high risk is slightly (but not significantly) higher with POD24 compared to RSF.

## 4. Discussion

In this study, we sought to compare the predictive performance of an RSF model versus a Cox model on datasets including traditional curated variables (i.e., patient demographics and disease-specific characteristics) along with higher-dimensional data (i.e., lab values and/or ICD diagnostic codes) from the EHRs of a population of patients with FL. We also compared performance of the RSF model against the most accurate known indicator of FL risk, POD24. Note that this comparison is inherently biased towards POD24 since our model considered information available at the time of L1 treatment initiation, while POD24, based on progression-free survival in the 24 months following L1 treatment initiation, incorporates information about the disease course a full two years after our model’s prediction is made.

We demonstrate that a limited set of EHR-extractable variables can provide similar performance to those generated from clinical trials [12]. While these limited EHR-derived variables would not necessarily provide a predictive gain over established clinical indices, they offer the advantage of being automatically extractable and computable, such that a modern EHR could simply provide them to the clinician without the need for the clinician to recall them, calculate them manually, and then include them in the chart. These prognostic factors would also be identifiable in the EHR from discrete structured data without needing to deploy information extraction or natural language processing approaches on unstructured data.

Our findings show that a broader set of data extracted from the EHR can improve prediction performance. However, not all data achieved such an effect. ICD codes added relatively little to prediction performance. On the other hand, lab values, available in EHRs but not other claims-based healthcare databases, substantially improved prediction. These results suggest that studies of the utility of EHR data in predicting clinical endpoints should emphasize EHR information that reflects the biology of patient and disease and deemphasize administrative data.

In addition, while our model included disease-specific variables commonly considered a reflection of disease risk (e.g., stage and grade), the variable importance analysis identified none of these variables as being among the most useful variables for prediction. Instead, the top variables reflected age, along with kidney, liver, and marrow function. This finding suggests that change in these variables may serve as the best indicator of disease risk, host fitness, and the host’s ability to tolerate the stresses introduced by the disease and its associated treatments. The correlation between age and survival probability in our model corroborates findings that a model including age in three categories (≤60 years old/61–70 years old/>70 years old) improves the predictive performance of FLIPI [12].

The best-performing combination of model and data we found, RSF applied to the curated + labs dataset, could not outperform POD24, although we found no significant difference between the AUCs of the two models. We report a relative risk for the predicted high- vs. low-risk groups using our best model of 4.39, lower than the previously reported risk ratio of POD24 vs. reference group of 6.44 [15]. However, our model offers the distinct advantage of using information available at the time of L1 treatment initiation, without the need to wait for two years while patients are observed for progression. An earlier prognosis would not only provide more information to patients about the potential impact of their disease on survival, it would also lead to significant differences in their management. Patients with high risk could be monitored more closely and would likely receive imaging surveillance more promptly if they developed symptoms that could indicate a recurrence. In addition, identification of high risk at the time of L1 initiation allows for adapting L1 to the disease risk, either by choosing a more aggressive treatment regimen and/or by adopting maintenance treatment (additional immunotherapy) following the completion of L1 treatment.

### Limitations

There are several limitations in this study. First, the majority of VHA patients are male, limiting generalizability of our results to a subset of the population of patients with FL. However, we implemented our methods in a nationwide cohort using structured EHR data commonly available in any EHR system, making it feasible to widely adopt our methods to automatically predict individual patient risk at the beginning of L1. Secondly, we limited our analysis to variables available in structured format; we did not include unstructured data in radiology reports, which might improve prognostic ability. With advances in precision medicine, genetic profiling and deep sequencing of tumors could likely become a widely adopted standard of care. Such information would probably improve prognosis as well. Lastly, we did not include deep learning approaches in our preliminary comparison of machine learning methods due to their computational cost and the need for specialized hardware.

## 5. Conclusions

RSF using EHR data achieved better performance than traditional prognostic variables. Our method sets the foundation for the incorporation of our algorithm into the EHR. It also allows for possible future scenarios to provide clinicians with an EHR-based tool which approximates the predictive ability of the most accurate known indicator, using information available 24 months earlier in the disease trajectory.

## Figures and Tables

**Table 1:** Baseline characteristics of patients by first-line treatment regimen.

	BR	RCHOP	RCVP	*p*-Value
N	120	235	168	
Sex = male * (%)	113 (94.2)	220 (93.6)	165 (98.2)	0.085
Race (%)				0.177
Hispanic	3 (2.5)	10 (4.3)	3 (1.8)	
Non-Hispanic Black	6 (5.0)	26 (11.1)	9 (5.4)	
Non-Hispanic White	109 (90.8)	194 (82.6)	153 (91.1)	
Other	2 (1.7)	5 (2.1)	3 (1.8)	
Disease stage (%)				0.254
II	12 (10.0)	42 (17.9)	20 (11.9)	
III	54 (45.0)	97 (41.3)	78 (46.4)	
IV	54 (45.0)	96 (40.9)	70 (41.7)	
Disease grade (%)				<0.001
1	38 (31.7)	55 (23.4)	70 (41.7)	
1–2	11 (9.2)	7 (3.0)	11 (6.5)	
2	58 (48.3)	76 (32.3)	69 (41.1)	
3	8 (6.7)	63 (26.8)	12 (7.1)	
3a	5 (4.2)	34 (14.5)	6 (3.6)	
Region of residence (%)				0.110
Midwest	33 (27.5)	60 (25.5)	38 (22.6)	
Northwest	17 (14.2)	26 (11.1)	36 (21.4)	
South	46 (38.3)	86 (36.6)	53 (31.5)	
West	24 (20.0)	63 (26.8)	41 (24.4)	
Pre-L1 CCI (mean [SD])	2.36 (2.56)	2.51 (2.58)	2.03 (2.33)	0.161
Age > 60 years at L1 (%)	85 (70.8)	149 (63.4)	117 (69.6)	0.259
Hemoglobin at L1 < 12 g/dL (%)	32 (26.7)	76 (32.3)	52 (31.0)	0.544
LDH at L1 > upper limit of normal	39 (32.5)	91 (38.7)	48 (28.6)	0.097
Days from diagnosis to L1 (mean [SD])	227.47 (321.72)	116.53 (314.30)	168.35 (328.04)	0.008

* The high percentage of male patients reflects the demographics of the Veterans Health Administration’s patient population. BR: bendamustine and rituximab; CCI: modified Charlson comorbidity index; L1: first-line; LDH: lactate dehydrogenase; RCHOP: rituximab, cyclophosphamide, doxorubicin, vincristine, and prednisone; RCVP: rituximab, cyclophosphamide, vincristine, and prednisone; SD: standard deviation.

**Table 2:** Cox and RSF model performance with different predictor datasets.

Model (AUC)	Curated (95% CI)	Curated + Labs (95% CI)	Curated + ICD (95% CI)	Curated + ICD + Labs (95% CI)
Cox (regularized Cox denoted by *)	0.64 (0.61–0.67)	0.61 (0.59–0.64) * 0.71 (0.69–0.73)	* 0.69 (0.67–0.71)	* 0.73 (0.70–0.75)
RSF	0.68 (0.65–0.70)	0.73 (0.71–0.75)	0.63 (0.61–0.65)	0.71 (0.63–0.79)

* Normal Cox model cannot handle high-dimensional data such as ICD codes; we provide performance measures of both normal and regularized Cox models for the curated + labs dataset for comparison. AUC: area under the receiver operating characteristic curve; CI: confidence interval; ICD: International Classification of Diseases diagnostic codes; RSF: random survival forest.

**Table 3:** Cox model performance with curated set and POD24.

Model (AUC)	Curated + POD24 (95% CI)
Cox	0.74 (0.71–0.77)

AUC: area under the receiver operating characteristic curve; CI: confidence interval; POD24: progression of disease within 24 months of starting first-line treatment.

**Table 4:** Relative risk for the predicted high- and low-risk groups.

Risk Group	N	Hazard Ratio (95% CI)	5-Year Overall Survival (95% CI)
**RSF Model**			
Low	62	1	0.83 (0.73–0.94)
High	43	4.39 (2.11–9.14)	0.44 (0.30–0.63)
**POD24 Model**			
Low	61	1	0.87 (0.78–0.98)
High	25	5.55 (3.27–9.35)	0.41 (0.26–0.68)

CI: confidence interval; POD24: progression of disease within 24 months of starting first-line treatment; RSF: random survival forest.

**Table 5:** Baseline characteristics of predicted high- and low-risk groups.

	Low-Risk	High-Risk	*p*-Value
N	62	43	
Sex = male * (%)	59 (95.2)	40 (93.0)	0.971
Race (%)			0.520
Hispanic	1 (1.6)	1 (2.3)	
Non-Hispanic Black	5 (8.1)	1 (2.3)	
Non-Hispanic White	52 (83.9)	40 (93.0)	
Other	2 (3.2)	0	
Unknown	2 (3.2)	1 (2.3)	
Disease stage (%)			0.503
II	8 (12.9)	5 (11.6)	
III	26 (41.9)	13 (30.2)	
IV	24 (38.7)	23 (53.5)	
Unknown	4 (6.5)	2 (4.7)	
Disease grade (%)			0.208
1	13 (21.0)	12 (27.9)	
1–2	3 (4.8)	0	
2	18 (29.0)	7 (16.3)	
3	8 (12.9)	7 (16.3)	
3a	8 (12.9)	3 (7.0)	
Unknown	12 (19.4)	14 (32.6)	
L1 treatment regimen (%)			0.483
BR	13 (21.0)	6 (14.0)	
RCHOP	25 (40.3)	22 (51.2)	
RCVP	24 (38.7)	15 (34.9)	
Region of residence (%)			0.789
Midwest	11 (17.7)	10 (23.3)	
Northwest	6 (9.7)	6 (14.0)	
South	17 (27.4)	8 (18.6)	
West	13 (21.0)	8 (18.6)	
Unknown	15 (24.2)	11 (25.6)	
Pre-L1 CCI (mean [SD])	2.37 (2.42)	2.93 (2.81)	0.284
Days from diagnosis to L1 (mean [SD])	135.31 (310.77)	102.49 (200.15)	0.543
Age > 60 years at L1 (%)	37 (59.7)	32 (79.1)	0.061
Hemoglobin at L1 (%)			<0.001
<12 g/dL	3 (4.8)	26 (60.5)	
≥12 g/dL	58 (93.5)	17 (39.5)	
Unknown	1 (1.6)	0	
LDH at L1 (%)			0.009
≤Upper limit of normal	47 (75.8)	21 (48.8)	
>Upper limit of normal	9 (14.5)	17 (39.5)	
Unknown	6 (9.7)	5 (11.6)	

* The high percentage of male patients reflects the demographics of the Veterans Health Administration’s patient population. BR: bendamustine and rituximab; CCI: modified Charlson comorbidity index; L1: first-line; LDH: lactate dehydrogenase; RCHOP: rituximab, cyclophosphamide, doxorubicin, vincristine, and prednisone; RCVP: rituximab, cyclophosphamide, vincristine, and prednisone; SD: standard deviation.

**Figure A1:**
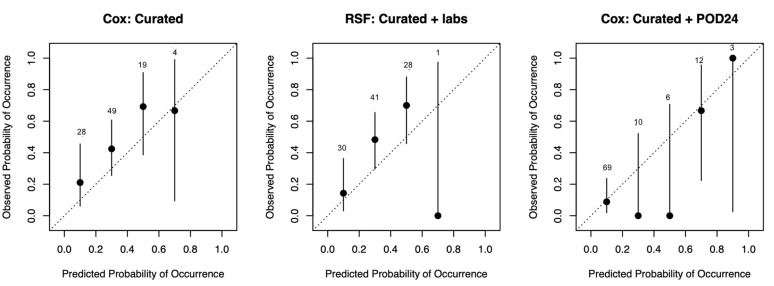
Calibration plots for Cox applied to curated set (AUC [95% CI]: 0.64 [0.61–0.67]), RSF models applied to the curated + labs set (AUC [95% CI]: 0.73 [0.71–0.75]), and Cox applied to curated + POD24 set (AUC [95% CI]: 0.74 [0.71–0.77]).

## Data Availability

The data that support the findings of this study are available through the Veterans Health Administration. Restrictions applying to the availability of these data were approved under the University of Utah Institutional Review Board #00083982 and the VA Salt Lake City Human Research Protection Program. Please contact the corresponding author for data inquiries.

## Appendix A. Clinical Rationale for Inclusion and Exclusion Criteria

Patients with Grade 3B and Stage I disease are treated differently: Stage I patients mostly receive radiation, while Grade 3B is thought to be a different clinical and biological entity whose treatments are more aligned with diffuse large B-cell lymphoma than FL. We excluded patients who receive single-agent rituximab since this therapy is less efficacious than the other three treatments and is typically given either in patients who do not meet treatment indication criteria or are too fragile to receive a more aggressive standard treatment. We also excluded maintenance rituximab since this is shown to prolong progression-free survival and would not allow for POD24 calculation, the latter reported mainly in patients who did not receive maintenance rituximab. Prior malignancy is a common exclusion criteria as a prior malignancy recurrence could lead to increased mortality independent from FL.

## Appendix B. RSF Parameter Tuning

In the training process, we used a grid search in order to find the optimal parameters;, i.e., an extensive grid search is performed with 5-fold cross-validation of each split. The parameters we tuned are: average number of observations in terminal nodes (nodesize), number of trees in the forest (ntree), and the maximum number of splits for continuous variables (nsplit). The tuning process is based on the C-index of cross-validation test results.

The parameters we specified using R code with the grid search are:

Nodesize: seq(100, 1000, by = 50)
Ntree: seq(1, ncol(train_data), length.out = 100)
Nsplit: c(1:9, seq(10, 100, by = 5))

## Appendix C. Tables and Figure

**Table A1:** Variables included in “labs” and “curated” datasets.

**Labs Set**
Alanine aminotransferase
Albumin
Alkaline phosphatase
Basophils
Basophils per 100 leukocytes
Bilirubin
Calcium
Carbon dioxide
Chloride
Creatinine
Eosinophils
Eosinophils per 100 leukocytes
Erythrocyte distribution width
Erythrocyte mean corpuscular hemoglobin
Erythrocyte mean corpuscular hemoglobin concentration
Erythrocyte mean corpuscular volume
Erythrocytes
Glomerular filtration rate per 1.73 square meters predicted
Glucose
Hematocrit
Lymphocytes
Lymphocytes per 100 leukocytes
Monocytes
Monocytes per 100 leukocytes
Neutrophils
Platelet mean volume
Platelets
Potassium
Protein
Sodium
Urea nitrogen
**Curated Set**
Sex
Race
Disease stage
Disease grade
Modified Charlson comorbidity index prior to first-line treatment
First-line treatment regimen
Age at first-line treatment initiation
Hemoglobin at first-line treatment initiation
Lactate dehydrogenase
Region of residence
Days from diagnosis to starting first-line treatment

**Table A2:** Cox model performance (AUC) using “curated” dataset with two-way interactions and restricted cubic spline.

Model	Curated (95% CI)
Cox	0.59 (0.56–0.61)

AUC: area under the receiver operating characteristic curve; CI: confidence interval.

**Table A3:** Cox model performance (AUC) on different datasets using multiple imputation instead of a random forest imputation algorithm.

Model	Curated (95% CI)	Curated + Labs (95% CI)	POD24 (95% CI)
Cox	0.64 (0.61–0.66)	0.64 (0.62–0.67)	0.75 (0.73–0.78)

AUC: area under the receiver operating characteristic curve; CI: confidence interval; POD24: progression of disease within 24 months of starting first-line treatment.

**Table A4:** Cox model performance (AUC) with two-way interactions using a stepwise variable selection approach with different datasets.

Model	Curated + Labs (95% CI)	Curated + ICD (95% CI)	Curated + ICD + Labs (95% CI)
Cox	0.64 (0.62–0.67)	0.57 (0.55–0.59)	0.59 (0.55–0.63)

AUC: area under the receiver operating characteristic curve; CI: confidence interval; ICD: International Classification of Diseases diagnostic codes.

**Table A5:** Number of times (out of 30 splits) that different variables were retained in the regularized Cox model after lasso regularization, with different datasets.

Labs		ICD		Labs + ICD	
Variable	Times Retained	Variable	Times Retained	Variable	Times Retained
Urea nitrogen	30	Age at L1	30	Age at L1	30
Age at L1 initiation	30	Hemoglobin *	30	Albumin *	30
Albumin	30	424.1	19	Urea nitrogen *	29
Erythrocytes	30	v45.81	19	Erythrocytes *	27
Chloride	16	362.02	18	v45.81	24
Protein	11	414.01	14	424.1	23
Calcium	10	250.80	13	v81.1	14
Lymphocytes	6	782.3	13	305.03	13
Carbon dioxide	5	v58.61	13	v58.61	13
Aspartate aminotransferase	4	v68.89	11	216.5	12
Potassium	4	305.03	10	414.01	12
Northeast US residence	4	443.9	7	v68.89	12
LDH	3	366.15	4	Potassium *	11
Sodium	3	523.42	4	285.8	10
Alanine aminotransferase	2	v58.83	3	362.02	10
Alkaline phosphatase	1	v76.43	3	250.80	9
Basophils	1	173.9	2	782.3	9
Bilirubin	1	244.9	2	362.05	6
Eosinophils	1	427.31	2	443.9	6
Hematocrit	1	721.3	2	Protein *	6
Neutrophils	1	v58.66	2	366.15	5
Platelets	1	250.00	1	427.31	5
RCVP as L1	1	295.32	1	v15.82	4
Male sex	1	300.02	1	v49.89	4
—	—	427.89	1	340.	3
—	—	428.0	1	371.5	3
—	—	715.00	1	785.2	3
—	—	721.2	1	v43.1	3
—	—	722.0	1	295.32	2
—	—	724.3	1	362.01	2
—	—	785.2	1	726.73	2
—	—	998.83	1	v16.1	2
—	—	LDH*	1	v58.83	2
—	—	v15.82	1	v76.43	2
—	—	v43.1	1	244.9	1
—	—	v65.19	1	266.2	1
—	—	v72.31	1	362.04	1
—	—	v72.6	1	366.16	1
—	—	—	—	369.9	1
—	—	—	—	427.9	1
—	—	—	—	523.42	1
—	—	—	—	586.	1
—	—	—	—	702.19	1
—	—	—	—	713.5	1
—	—	—	—	721.2	1
—	—	—	—	721.3	1
—	—	—	—	726.32	1
—	—	—	—	786.9	1
—	—	—	—	793.99	1
—	—	—	—	998.83	1
—	—	—	—	Aspartate aminotransferase *	1
—	—	—	—	Chloride *	1
—	—	—	—	E878.2	1
—	—	—	—	Glucose *	1
—	—	—	—	v43.3	1
—	—	—	—	v65.19	1
—	—	—	—	72.31	1

* denotes imputed result. ICD: International Classification of Diseases diagnostic codes. L1: first-line treatment; LDH: lactate dehydrogenase; RCVP: rituximab, cyclophosphamide, vincristine, and prednisone.
